# HiPorfin photodynamic therapy for vaginal high-grade squamous intraepithelial lesion

**DOI:** 10.1007/s00404-024-07600-4

**Published:** 2024-06-20

**Authors:** Yu Liu, Ruifang Wu, Changzhong Li, Lvfang Duan, Lihui Wei, Ruizhen Li

**Affiliations:** 1 Department of Obstetrics and Gynecology, Peking University Shenzhen Hospital, Shenzhen, China; 2 Institute of Obstetrics and Gynecology, Shenzhen Peking University-Hong Kong University of Science and Technology (PKU-HKUST) Medical Center, Shenzhen, China; 3 Shenzhen Key Laboratory on Technology for Early Diagnosis of Major Gynecologic Diseases, Peking University Shenzhen Hospital, Shenzhen, China; 4 Director of Sanming Project of Medicine of Department of Obstetrics and Gynecology, Peking University Shenzhen Hospital, Shenzhen, China; 5 Department of Obstetrics and Gynecology, Peking University People’s Hospital, Beijing, China

**Keywords:** Photodynamic therapy, Vaginal intraepithelial neoplasia, Vaginal high-grade squamous intraepithelial Lesion, Efficacy, Safety

## Abstract

**Purpose:**

We aimed to evaluate the efficacy and safety of HiPorfin-photodynamic therapy (PDT) in women with vaginal high-grade squamous intraepithelial Lesion (HSIL).

**Methods:**

Retrospective analysis of eighteen patients with vaginal HSIL received HiPorfin-PDT between June 2019 and May 2023. Illumination with a 630-nm laser light was applied to the lesions 48–72 h after intravenous injection of 2 mg/kg HiPorfin^®^. The light dose to the lesions was 150 J/cm^2^.

**Results:**

The mean age of the 18 patients was 45.8 years (range, 24 to 63). The complete response (CR) rate was 66.7% (12/18), 83.3% (15/18) and 83.3% (15/18) at 3, 6 and 12 months after PDT, respectively. Patients who achieved CR showed no signs of recurrence during long-term follow-up. There were three cases of persistent disease showing partial response (PR) and the lesion area was significantly reduced more than 50%. One patient with persistent disease then underwent thermocoagulation one time and subsequently showed no evidence of HSIL. Pre-treatment, 100% (18/18) patients were high-risk human papilloma virus (HR-HPV)-positive. HPV eradication rate was 16.7% (3/18), 22.2% (4/18) and 44.4% (8/18) after PDT at 3, 6 and 12 months, respectively. Before treatment, liquid-based cytology test ≥ atypical squamous cells of undetermined significance (ASCUS) was 94.4% (17/18). Negative conversion ratio of cytology was 47.1% (8/17), 52.9% (9/17) and 76.5% (13/17) at 3, 6 and 12 months, respectively. There were no serious adverse effects during and after PDT.

**Conclusions:**

HiPorfin-PDT may be an effective alternative treatment for vaginal HSIL for organ-saving and sexual function protection.

## Take-home message

**Table:** 

Due to increased rate of vaginal high-grade squamous intraepithelial Lesion, effective and noninvasive treatments for vaginal HSIL have great clinical needs. Our study showed complete response rate was 83.3% at 6 months after HiPorfin-photodynamic therapy which may be an effective alternative treatment for vaginal HSIL for organ-saving and sexual function protection.	

## Introduction

Vaginal intraepithelial neoplasia (VAIN) is a rare intraepithelial lesion of female lower genital tract closely related to HR-HPV and only accounts for approximately 0.4% of intraepithelial lesions in the female lower genital tract [1]. VAIN is rarely appearing alone, but often exhibits concomitant or previous cervical intraepithelial neoplasia (CIN) or vulvar intraepithelial neoplasia (VIN) [2, 3]. VAIN is mostly a continuation of CIN and often multifocal, mainly occurring in the upper 1/3 of the vagina. Most of VAIN symptoms are atypical, with only a few showing bleeding, abnormal vaginal secretion or itching [4]. Diagnosis, treatment, and follow-up of VAIN are difficult and insufficient due to the special anatomical structure of vagina. However, there is increased rate of VAIN in recent years, with 0.3–0.5 cases per 100,000 women worldwide [5], which has been associated with as a result of increased cervical cancer screening, improved colposcopy technique, and increased standard follow-up after LEEP or hysterectomy for CIN or cervical cancer. Recurrence of VAIN is common and requires repeatedly treatment with long-term follow-up. Vaginal squamous intraepithelial lesion had been classified into two levels as low-grade squamous intraepithelial lesion (LSIL) including VAINI and HSIL, including VAINII or VAINIII [6]. Active medical intervention is recommended for HSIL because of its high risk of progression to malignant tumor [7, 8]. Approximately 10% of VAINIII progressed to invasive carcinoma of vagina [9].

Currently, the recommended treatment methods for vaginal HSIL include local drug treatment, carbon dioxide (CO2) laser, surgical excision and vaginal brachytherapy [10–14]. Each of these therapies has its advantages and disadvantages. For instance, drug treatment such as 5-fluorouracil (5-FU) or imiquimod is simple, however, is associated with high recurrence rates and adverse effects such as irritation, pain, and dyspareunia. Surgery with high cure rate is difficult for large and multi-site vaginal lesions and partial vaginectomy might cause vaginal stenosis, shortening, scarring, dyspareunia and peripheral organs damage which was unacceptable for most patients especially among young women. CO2 laser which is the most common contemporary treatment for vaginal HSIL damages the vaginal mucosa and is simple to operate [15–17]. However, the depth of CO_2_ laser treatment is not enough and repeated CO_2_ laser treatments can cause local adhesions, perforations and vaginal scarring. Adhesion renders any remaining or recurrent lesions difficult to remove which may lead to further progression of disease. What is more, CO_2_ laser is not appropriate for vaginal vault lesions because of its limited access. Vaginal brachytherapy considered for elderly patients with recurrent is an effective and safe treatment, however, it should not be used as a first line treatment for vaginal HSIL, because of the topical damage that causes to blood vessels and connective tissue, which limits any future radiotherapy and surgical procedures for recurrences. Meanwhile, vaginal brachytherapy often results in vaginal stenosis or sexual dysfunction and is not appropriate for young patients due to the risks of premature ovarian failure and vaginal constriction. There is no guideline to the optimal treatment of vaginal HSIL. Therefore, more effective or noninvasive treatments for vaginal HSIL have great clinical needs. PDT might be the most adherent to the conditions mentioned above.

PDT as a modern, non-invasive and highly selective treatment involves photosensitizer, light irradiation, and oxygen. The photosensitizer selectively concentrating at abnormal cells is activated by light at a specific wavelength and transfers its energy to molecular oxygen to produce singlet oxygen, resulting in a series of photochemical reactions which lead to necrosis or apoptosis of the target cells without injuring the normal tissues. It is successfully used in many fields of medicine [18]. PDT is based on the systemic or topical application of photosensitizers. Because the surfaces of VAIN lesions are usually hyperkeratotic, local PDT such as 5-aminolevulinic acid (ALA) PDT may face physical limitations. Meanwhile, due to the limited penetration of the drug and the depth of illumination, treatments such as CO_2_ laser are required before local PDT. Systemic PDT with only once treatment showed exciting results [19]. In our research, we performed PDT with systemic photosensitizer of HiPorfin^®^.

HiPorfin^®^ which can be applied in both precancerous lesions and cancers is the only one of hematoporphyrin derivatives (HpD) going through formal clinical trials and approved for oncological indications by Chinese State Food and Drug Administration (SFDA) in 2001 [20]. Other foreign intravenous photosensitizers are still not approved by Chinese SFDA. Some reports has confirmed its efficacy [21–23]. Our previous research showed HiPorfin-PDT for cervical HSIL at childbearing age was encouraging and allow patients to have full-term delivery [24, 25]. HiPorfin-PDT has advantages for large and multifocal lesions.

HiPorfin-PDT is easy to use and learning curve is short. The operating physician should be trained in photodynamic dosimetry and gynecological tumor photodynamic therapy for about two weeks. Most HiPorfin-PDT only requires once treatment time. For patients with a body weight of 50 kg, the cost of once treatment session with HiPorfin^®^ is about 15,000 RMB which is about 3000–4000 RMB cheaper than the cost of six treatment sessions with ALA. Furthermore, patient’s time costs are significantly saved with HiPorfin-PDT.

Up to now, there are no relevant reports in HiPorfin-PDT for vaginal HSIL currently and our research explements the gap. The objective of this study was to evaluate the clinical effectiveness and safety of HiPorfin^®^ for vaginal HSIL, with HR-HPV infection.

## Materials and methods

A total of 18 patients aged 24–63 years with morphologically proven diagnosis of vaginal HSIL were enrolled in this study from June 2019 to May 2023. None of the patients was lost. The patients were informed about the main objective of the study, process, side effects and the options of conventional treatments including local drug treatment, CO_2_ laser, surgery and intravaginal radiation therapy and provided their written informed consent to participate in this study.

The inclusion criteria were as follows: vaginal HSIL. The exclusion criteria were as follows: possibility of invasive carcinoma of vagina, high-grade CIN or high-grade VIN by colposcopy or histology examination; patients allergic to HiPorfin^®^ or known porphyria; pregnancy, lactation period or menstrual period; patients with obvious impaired heart, liver, and kidney functions; patients with coagulation dysfunction, serious uncontrolled medical complications, acute inflammatory period; antithrombotic or antiplatelet agglutination drugs were being used in high doses.

Initial clinical examination before treatment included bacteriologic examination of vaginal and cervical secretion, HPV test (Cobas^®^ 4800), liquid-based cytology test (AutoCyte^®^ thin-layer cytology test (TCT)), colposcopy, pathological examination of biopsy samples, blood routine examination, coagulation function, hepatorenal function, blood human chorionic gonadotropin (HCG), electrocardiogram, et al. Colposcopy was performed by experienced colposcopists.

**Fig. 1:**
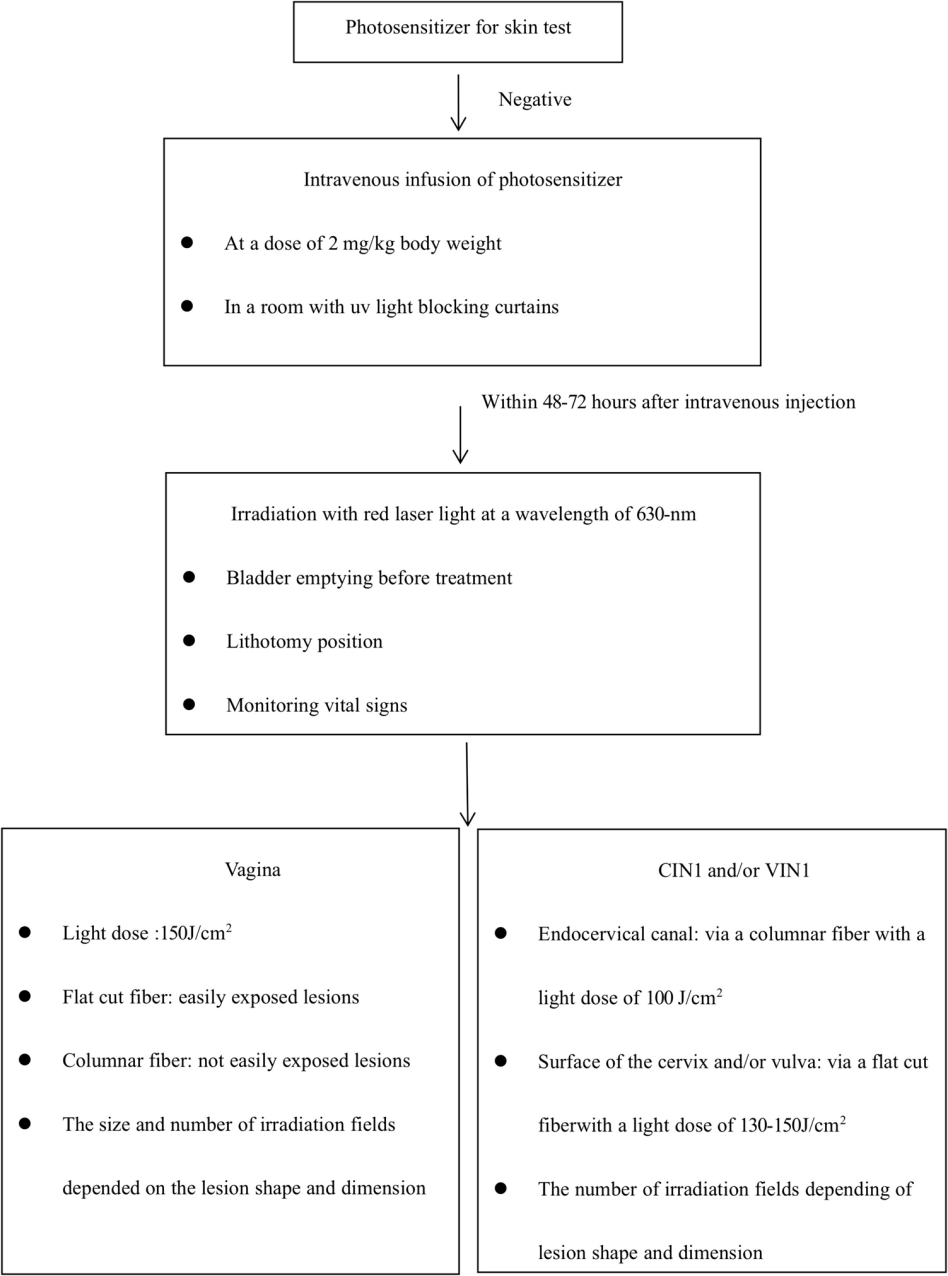
Procedure of photodynamic therapy

Photosensitizer (HiPorfin^®^; Chongqing Milelonge Biopharmaceutical Co., Ltd, China) was injected intravenously at a dose of 2 mg/kg in a room blocked by UV light after hospitalization, 48–72 h prior to laser light application. Patients should stay away from sunlight while in hospital. HiPorfin-PDT only requires once treatment time. Bladder emptying before treatment. Monitoring vital signs was required during treatment. The patients were put at a lithotomy position, disinfected and draping was performed. Colposcopy was performed before each PDT to assess the locations, size, and number of the lesions after the application of acetic acid followed by iodine test. The vaginal lesions were then illuminated using a red-light (PDT630 semiconductor laser treatment machine: Shenzhen Laser Medical Technology Co., Ltd, China) at a wavelength of 630 nm and a light dose of 150 J/cm^2^ with the flat cut fiber and/or columnar fiber (Shenzhen Laser Medical Technology Co., Ltd, China) which overlapped the lesions for at least 0.5 cm at any edge. The size and number of irradiation fields depended on the lesion shape and dimension. If patients with CIN1 and/or VIN1 combined, endocervical canal was irradiated via a columnar fiber with a light dose of 100 J/cm^2^. The surface of the cervix and/or vulva was irradiated via a flat cut fiber with a light dose of 130–150 J/cm^2^ (Fig. 1).

All patients were educated to wear sun-shield items to protect the skin and eyes from exposure to direct sunlight for 4–6 weeks to avoid systemic side effects because the photosensitizer can make the skin and eyes sensitive to light (mainly sunlight) for approximately 4–6 weeks after injection. Nevertheless, daily life such as watching mobile phone, using computer and watching TV was all unaffected. Patients were asked to use contraception until getting the results of 3 months follow-up.

The efficacy of PDT was evaluated by gynecological examinations in 3, 6 and 12 months and then every 12 months afterwards. The examinations included: clinical examination, HPV test, cytology test, colposcopy and biopsy. In terms of remission, no definitive criteria have been determined in PDT. The postoperative assessment of PDT was based on postoperative cytology, colposcopy and biopsy using the following common criteria: complete response (CR) (cytology, colposcopy and biopsy showed no high-grade lesions); partial response (PR) (at least 50% reduction of HSIL area); stabilization (less than 50% reduction of HSIL area); progression (biopsy pathology confirmed invasive carcinoma). Eradication rate of HR-HPV and negative conversion ratio of cytology were assessed. PDT-related adverse events such as vaginal discharge, pelvic pain and photosensitivity, et al. were also recorded in accordance with Common Terminology Criteria for Adverse Events (CTCAE) Ver. 5.0. SPSS 27.0 software was used for all statistical analyses.

## Results

Of the 18 patients in this study, the mean age was 45.8 ± 14.2 years old (range: 24–63) and the median duration was 22.5 months (range: 7–54 months). Five (27.8%, 5/18) patients were nulliparous and four (22.2%, 4/18) were under age 30. Seven patients underwent hysterectomy and two patients underwent a loop electrosurgical excision procedure (LEEP) for cervical HSIL before. One patient underwent hysterectomy due to adenomyosis. For the remaining patients who did not have surgery on cervix or uterus, two patients found vaginal HSIL due to irregular vaginal bleeding and the rest patients were diagnosed this disease due to abnormal cervical cancer screening results of routine examination. Pre-treatment, 100% (18/18) of patients were infected with HR-HPV and TCT ≥ ASCUS was 94.4% (17/18). Meanwhile, 88.9% (16/18) cases had multifocal lesions (Table 1).

**Table 1:** Patients’ baseline characteristics

Patient no	Age (years)	Duration (months)	Nullipara	Previous cervical/uterine diseases	Previous cervical/uterine treatment	HR-HPV	TCT	Number of lesions
1	62	54	No	HSIL	LEEP	Positive	ASCUS	Multifocal
2	53	52	No	HSIL	Hysterectomy	Positive	ASCUS	Multifocal
3	33	47	No	None	None	Positive	ASCUS	Multifocal
4	35	32	Yes	None	None	Positive	LSIL	Multifocal
5	62	32	No	None	None	Positive	ASC-H	Unifocal
6	49	25	No	HSIL	Hysterectomy	Positive	LSIL	Multifocal
7	53	27	No	Adenomyosis	Hysterectomy	Positive	LSIL	Multifocal
8	60	23	No	HSIL	Hysterectomy	Positive	HSIL	Multifocal
9	28	22	No	HSIL	Hysterectomy	Positive	ASCUS	Multifocal
10	29	19	Yes	None	None	Positive	NILM	Multifocal
11	59	16	No	HSIL	Hysterectomy	Positive	ASCUS	Multifocal
12	48	10	No	HSIL, Atypical endometrial hyperplasia	Hysterectomy	Positive	LSIL	Multifocal
13	63	7	No	HSIL^a^	Hysterectomy	Positive	LSIL	Multifocal
14	54	22	No	LSIL	None	Positive	LSIL	Multifocal
15	24	12	Yes	LSIL	None	Positive	ASCUS	Unifocal
16	54	13	No	LSIL	None	Positive	ASCUS	Multifocal
17	34	10	Yes	HSIL	LEEP	Positive	LSIL	Multifocal
18	24	35	Yes	LSIL	None	Positive	ASCUS	Multifocal

*HR-HPV* high-risk human papilloma virus, *TCT* thin-layer cytology test, *LEEP* loop electrosurgical excision procedure, *NILM* nonintrusive load monitoring, *ASCUS* atypical squamous cell of undetermined significance, *LSIL* low-grade squamous intraepithelial lesion, *ASC-H* atypical squamous cells-cannot exclude high-grade lesions, *HSIL* high-grade squamous intraepithelial lesion

^a^The patient underwent LEEP for cervical HSIL 2 years ago and results of follow-up showed vaginal HSIL with cervical CINI and VINI combined

**Fig. 2:**
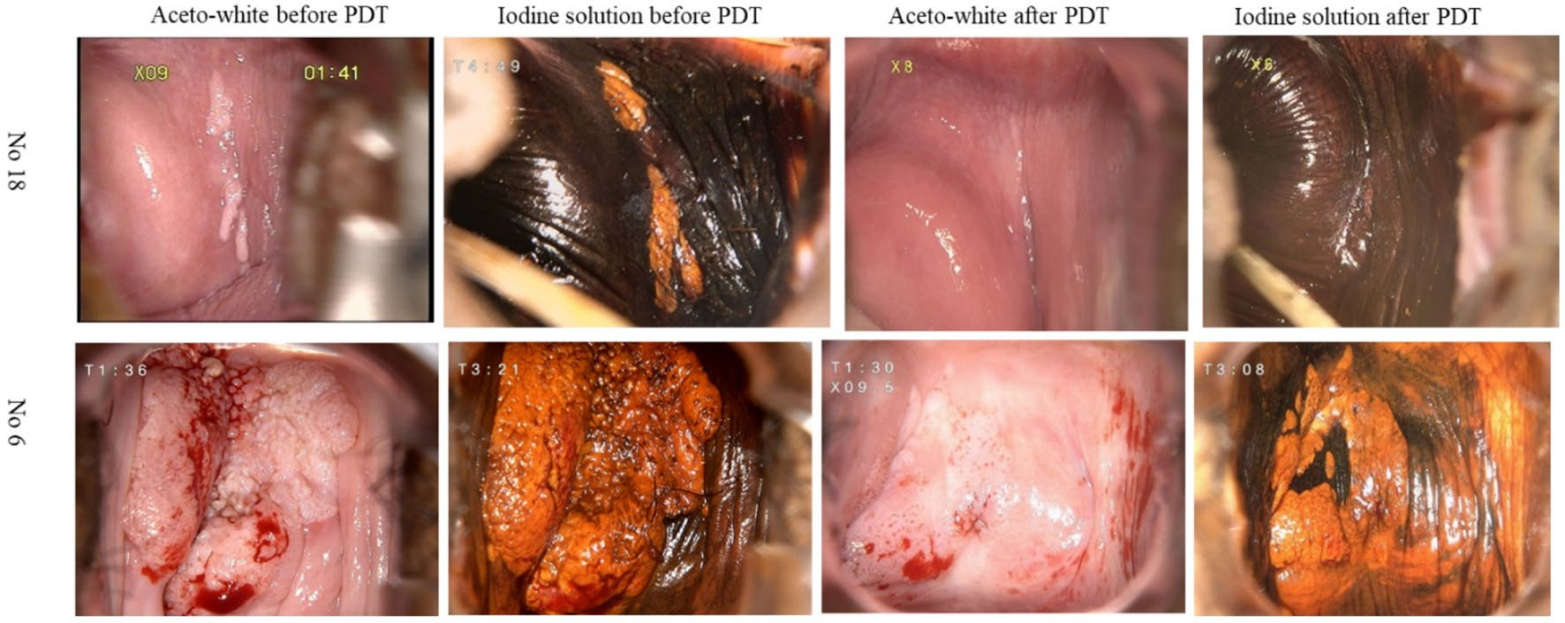
Comparison of colposcopy before and after PDT from Case 18, aged 24 years (upper panel) and Case 6, aged 49 year (lower panel). The first column reflects the aceto-white dysplastic lesion areas before PDT. The second column depicts atypical epithelium after use of iodine solution before PDT. The third column reflects the aceto-white dysplastic lesion areas after PDT. The last column depicts atypical epithelium after use of iodine solution after PDT in the same patient

Negative conversion ratio of cytology was 47.1% (8/17), 52.9% (9/17) and 76.5% (13/17) at 3, 6 and 12 months, respectively. The total HPV clearance rate which meant the previously infected HPV subtypes disappeared was 16.7% (3/18), 22.2% (4/18) and 44.4% (8/18) after PDT at 3, 6 and 12-months follow-up, respectively. Meanwhile, CR rate was 66.7% (12/18), 83.3% (15/18) and 83.3% (15/18) at 3, 6 and 12 months after PDT, respectively (Table 2). During the long-term follow-up, patients with CR did not experience any recurrence. According to pathologic findings of colposcopic biopsy after treatment, PDT was ineffective in three patients with PR, all of whom had HPV 16 infection and multifocal lesions. One patient (no.4) with lesions on the vaginal vault and vaginal wall then underwent thermocoagulation one time and subsequent results of follow-up showed no evidence of HSIL. Two participants (no.6 and 8) with history of hysterectomy due to cervical HSIL had lesions on the vaginal stump and vaginal wall and the lesions significantly were smaller than that before PDT (Table 3, Fig. 2).

**Table 2:** Treatment outcome of the PDT in the vaginal HSIL

	Percentage
HPV eradication rate	
3 months	16.7% (3/18)
6 months	22.2% (4/18)
12 months	44.4% (8/18)
Negative conversion ratio of cytology	
3 months	47.1% (8/17)
6 months	52.9% (9/17)
12 months	76.5% (13/17)
Cure efficacy at 3 months	
CR	66.7% (12/18)
PR	33.3% (6/18)
Stabilization	0.0% (0/18)
Progression	0.0% (0/18)
Cure efficacy at 6 months	
CR	83.3% (15/18)
PR	16.7% (3/18)
Stabilization	0.0% (0/18)
Progression	0.0% (0/18)
Cure efficacy at 12 months	
CR	83.3% (15/18)
PR	16.7% (3/18)
Stabilization	0.0% (0/18)
Progression	0.0% (0/18)

*PDT* photodynamic therapy, *HSIL* high-grade squamous intraepithelial lesion, *HPV* human papilloma virus, *CR* complete response, *PR* partial response

**Table 3:** Characteristics of three patients with PR

Patient no	Age	HR-HPV	TCT	Number of lesions	Lesion location	History
4	35	16、68	LSIL	Multifocal	Vaginal vault + vaginal wall	No
6	49	16	LSIL	Multifocal	Vaginal stump + vaginal wall	Hysterectomy due to cervical HSIL
8	60	16	HSIL	Multifocal	Vaginal stump + vaginal wall	Hysterectomy due to cervical HSIL

*PR* partial response, *TCT* thin-layer cytology test, *HR-HPV* high-risk human papilloma virus, *LSIL* low-grade squamous intraepithelial lesion, *HSIL* high-grade squamous intraepithelial lesion

The procedure was tolerable during and after PDT. No severe adverse events were observed. The most common adverse events were increased vaginal discharge and mild (grade1) pelvic pain. Mild photosensitivity such as mild edema and burning sensation in the exposed parts was identified in 11.1% (2/18) of patients due to lack of sunblock education. And the allergic symptoms resolved with loratadine and ice with 7–10 days after guidance.

## Discussion

The diagnosis of vaginal HSIL has increased in recent decades, however, the true prevalence of vaginal HSIL may be underreported for several reasons: (1) cytology may fail to adequately sample the vaginal lesions; (2) special anatomic structure of vagina and lesion concealment without complete exposure; (3) biopsies may be too superficial, lacking the necessary vertical depth to accurately assess the lesion; (4) excessive secretion after treatment was not cleared promptly, leading to adhesions that can interfere with subsequent follow-up. Given these challenges, VAIN requires increased vigilance alongside the diagnosis and treatment of cervical lesions. Continuous monitoring through cytology and/or HPV testing, as well as thorough colposcopy performed by an experienced specialist, is essential for accurate diagnosis and effective management.

Age has been identified as a risk factor for VAIN, with postmenopausal women facing a 2.09 times higher risk for developing VAIN compared to premenopausal women [3]. The median age for VAIN incidence is reported to be 50 years old [26]. However, in this study, the age of patients with vaginal HSIL was slightly lower, at 45.8 ± 14.2 years (ranging from 24 to 63 years). Young patients have a stronger desire to preserve tissue integrity and function, leading them to opt for conservative management approaches over surgical interventions.

HPV infection, along with a history of CIN and cervical carcinoma are significant risk factors for the development of VAIN [3, 27, 28]. Studies indicates that the incidence of vaginal HSIL significantly increases following a hysterectomy for CIN or cervical cancer, with most cases occurring within 2 years post-surgery [29]. Previous LEEP or hysterectomy makes it more challenging to the follow-up colposcopy to see these lesions, biopsy the lesions and treat the patients. In this paper, 50% (9/18) patients had undergone hysterectomy or LEEP for cervical HSIL before receiving treatment for vaginal HSIL. 66.7% (6/9) of the patients were diagnosed with vaginal HSIL within 2 years after their operation, aligning with previous findings. This correlation may be attributed to the fact that hysterectomy does not eradicate HPV infection, which may lead to vaginal HSIL. In addition, persistent HR-HPV infection is also associated with the occurrence of VAIN [3, 30, 31]. HPV16 is the most prevalent infection, especially in patients with VAINII/III [31–33]. So et al. found that 74.3% of VAINI, 85.7% of VAINII and 100% of VAINIII lesions were associated with HR HPV DNA [34]. In our study, all patients were infected with HR-HPV and the infection rate of HPV16 was 61.1% (11/18). Previous researches have showed the HPV remission rates between 38.2% and 77.2% [11, 17]. It is common for VAIN patients who have undergone hysterectomy for CIN or cervical cancer to retain the same HPV subtypes as before surgery. A prolonged history of HPV infection makes it difficult to clear the virus. The HPV clearance rate of 41.9% observed in our study is considered acceptable. However, five cases were followed up for less than 12 months. Larger sample size and longer follow-up time are necessary to validate the long-term efficacy of HPV clearance.

There are multiple treatment options for managing vaginal HSIL, yet patients with vaginal HSIL face a high risk of recurrence and progression. The recurrence rates of surgical resection, laser therapy, drug therapy, and observation were 38, 43, 75, and 50%, respectively [27]. Currently, there are no clinical guidelines or a universally accepted treatment protocol for vaginal HSIL treatment. The ideal treatment should have high rate of CR, be noninvasive or minimally invasive, preserve normal anatomical structures, be repeatable without causing cumulative toxicity, and be effective for massive lesion and multifocal lesions. PDT as a highly selective therapeutic approach hardly affects the structure and function of surrounding normal tissues [35]. Among all available treatments, PDT most closely aligns with these criteria. PDT is based on the systemic or topical application of photosensitizers.

Second generation topical photosensitizers, such as ALA has the advantage of not needing to avoid sunlight, however, ALA's superficial penetration may not adequately reach all of the vaginal HSIL which needs multiple treatment sessions. The CR rate of ALA-PDT varies widely, from 7.1 to 88.89%, with recurrence rates ranging between 11.36 and 20.0% [36–38]. Meanwhile, treatments like CO_2_ laser are frequently required before PDT due to the limited penetration of the drug and the depth of illumination [39, 40]. However, repeated CO_2_ laser treatments can cause local adhesions affecting subsequent follow-up. In contrast, HiPorfin^®^ PDT typically requires only a single treatment session. Furthermore, the cure rate of HiPorfin^®^ PDT was encouraging even though half of participants had history of hysterectomy or LEEP due to cervical HSIL and most had multifocal lesions. Moreover, patients with CR experienced no recurrence during the long-term follow-up.

In our study, CR rate was 83.3% (15/18) at both 6 and 12 months with no recurrence which was higher than those reported in previous studies [8, 12, 16, 27]. This CR rate is higher than those reported in previous studies. We attribute the high CR rate to two main factors: (1) Thorough colposcopic assessment: the location and distribution of lesions were comprehensively evaluated by colposcopy before PDT; (2) Adequate treatment application: We used appropriate fibers to irradiate lesions based on their position. Flat cut fibers were applied to lesions that were easily exposed, while columnar fibers were used for lesions that were more difficult to expose. Analysis of clinical characteristics of three patients with PR revealed that all three patients (100.0%) had multifocal lesions. Skin fold is a negative factor in the PDT of VAIN. For lesions located in the vaginal fold, stumps or vaults, it is crucial to ensure full exposure of the vagina, using tools if necessary, to facilitate effective light irradiation and maintain the quality of treatment. Meanwhile, 100.0% (3/3) cases had HPV 16 infection and 66.7% (2/3) cases had previously undergone a hysterectomy for cervical HSIL before. These factors may have contributed to the suboptimal treatment outcomes observed in these patients.

In this study, we demonstrated that HiPorfin-PDT was an effective and safe treatment option for patients with vaginal HSIL, particularly for those with multifocal and massive lesions. Therefore, HiPorfin–PDT in vaginal HSIL deserves further research. However, the study had following limitations: (1) single-arm trial design, (2) lacked a control group, (3) small sample size. Thus, prospective randomized controlled studies with multicenter, lager sample size and extended follow-up time are necessary to further validate the efficacy and recurrence rate of HiPorfin-PDT. In addition, being 45 years of age or older, HR-HPV infection (especially HPV 16), having a history of LEEP or hysterectomy for CIN or cervical cancer, having lesions located at vaginal vault or stump, presenting with multiple lesions and massive lesions need more attentive follow-up care after HiPorfin-PDT.

## Data Availability

The data used in this study will be available upon reasonable request from the corresponding author, Ruizhen Li.
